# Genome Informatics 2016

**DOI:** 10.1186/s13059-016-1135-5

**Published:** 2017-01-16

**Authors:** Davide Chicco, Michael M. Hoffman

**Affiliations:** 1 Princess Margaret Cancer Centre, Toronto, Canada; 2 Department of Medical Biophysics, University of Toronto, Toronto, Canada; 3 Department of Computer Science, University of Toronto, Toronto, Canada

## Abstract

A report on the Genome Informatics conference, held at the Wellcome Genome Campus Conference Centre, Hinxton, United Kingdom, 19–22 September 2016.

## Personal and medical genomics

Several talks covered systems and new technologies that clinicians, patients, and researchers can use to understand human genomic variation. Jessica Chong (University of Washington, USA) described MyGene2 (http://mygene2.org), a website that allows families to share their de-identified personal data and find other families with similar traits. Jennifer Harrow (Illumina, UK) discussed using BaseSpace (https://basespace.illumina.com/) for the analysis of clinical sequencing data. Deanna Church (10x Genomics, USA) presented Linked-Reads, a technology that makes it easier to find variants in less accessible genomic regions such as the *HLA* locus. Several presenters showed new methods to identify the functional effects of sequence variants. Konrad Karczewsky (Massachusetts General Hospital, USA) presented the Loss Of Function Transcript Effect Estimator (LOFTEE, https://github.com/konradjk/loftee). LOFTEE uses a support vector machine to identify sequence variants that significantly disrupt a gene and potentially affect biological processes. Martin Kircher (University of Washington, USA) discussed a massively parallel reporter assay (MPRA) that uses a lentivirus for genomic integration, called lentiMPRA [3]. He used lentiMPRA to predict enhancer activity, and to more generally measure the functional effect of non-coding variants. William McLaren (European Bioinformatics Institute, UK) presented Haplosaurus, a variant effect predictor that uses haplotype-phased data (https://github.com/willmclaren/ensembl-vep).

Two presenters discussed genome informatics approaches to the analysis of cancer immunotherapy response. Meromit Singer (Broad Institute, USA) performed single-cell RNA profiling in dysfunctional CD8^+^ T cells. She identified metallothioneins as drivers of T cell dysfunction and revealed novel sub-populations of dysfunctional T cells [4]. Christopher Miller (Washington University, St Louis, USA) tracked the response to cancer immunotherapy in the genome of patients [5].

In a keynote lecture, Elaine Mardis (Washington University, St Louis, USA), described computational tools and databases created to collect and process cancer-specific mutation datasets. A substantive increase in the amount of clinical sequencing performed as part of cancer diagnosis and treatment necessitated the development of these tools. She emphasized the shift in categorization of cancers—previously oncologists classified cancers by tissue, but increasingly they classify cancers by which genes are mutated. Mardis suggested that we should instead describe cancers by the affected metabolic and regulatory pathways, which can provide insight even for previously unseen disruption. This disruption can be genetic mutations, but it can also manifest as other changes to cellular state, which must be measured with other techniques, such as RNA-seq. The tools Mardis described help interpret the mutations identified by sequencing. These include the Database of Curated Mutations (DoCM). She also described Personalized Variant Antigens by Cancer Sequencing (pVAC-seq), a tool for identifying tumor neoantigens from DNA-seq and RNA-seq data. She also described Clinical Interpretations of Variants in Cancer (CIViC), a platform for crowd-sourcing data on clinical consequences of genomic variants. CIViC has 1565 evidence items describing the interpretation of genetic variants, and Mardis announced a forthcoming Variant Curation Hackathon to identify more.

## Variant discovery and genome assembly

Several speakers presented tools and methods about analysis of genome assemblies and exploration of sequence variants. Jared Simpson (Ontario Institute for Cancer Research, Canada) started the second session with an overview of base calling for Oxford Nanopore sequencing data and his group’s contribution to this field, Nanocall (http://github.com/mateidavid/nanocall). Simpson also discussed Nanopolish, which can detect 5-methylcytosine from Oxford Nanopore sequencing data directly, without bisulfite conversion. Kerstin Howe (Wellcome Trust Sanger Institute, UK) presented her work with the Genome Reference Consortium on producing high quality assemblies for different strains of mouse and zebrafish. Ideally, future work will integrate graph assemblies. Frank Nothaft (University of California, Berkeley, USA) described ADAM (https://github.com/bigdatagenomics/adam), a library for distributed computing on genomics data, and Toil, a workflow management system. These systems are about 3.5 times faster than standard Genome Analysis Toolkit (GATK) pipelines.

Some presenters discussed genome assembly tools and datasets which might be utilized by the wider community. Andrew Farrell (University of Utah, USA) introduced RUFUS (https://github.com/jandrewrfarrell/RUFUS), a method for efficiently detecting de novo mutation using k-mer counting instead of reference-guided alignment. Alicia Oshlack (Murdoch Childrens Research Institute, Australia) presented the SuperTranscript model for enhancing transcriptome visualization (https://github.com/Oshlack/Lace/wiki). Jouni Sirén (Wellcome Trust Sanger Institute, UK) presented a method to index population variation graphs using FM-index [6]. His new package, GCSA2 (https://github.com/jltsiren/gcsa2), provides a toolkit to work with variation graphs. Birte Kehr (deCODE Genetics, Iceland) introduced a whole-genome sequencing dataset of approximately 15,000 Icelanders comprising approximately 4000 variants from non-repetitive sequences not in the reference assembly [7]. Giuseppe Narzisi (New York Genome Center, USA) presented Lancet, software to find somatic variants using localized colored De Bruijn graphs.

In a keynote lecture, Richard Durbin (Wellcome Trust Sanger Institute, UK) discussed genome reference assemblies and the pitfalls of using a single flat reference sequence. Genomicists use the reference genome for mapping sequencing reads, as a coordinate system for reporting and annotation, and as a framework for describing known variation. While the reference genome makes many analyses simpler, it biases these analyses towards what is previously seen. Durbin briefly discussed the advantages of the newest human reference assembly, GRCh38, which fixes many previous problems and includes alternate loci to capture complex genetic variation. But to more effectively work with this variation, Durbin said we need to switch from a flat reference to a “pan-genome” graph that includes much known variation [8]. To do this, we will need a new ecosystem of graph genome file formats and analysis software. Durbin discussed the work of the Global Alliance for Genomics and Health to evaluate proposed systems for working with graph genomes.

## Epigenomics and the non-coding genome

Speakers described new methods for epigenomic data, such as DNase-seq (deoxyribonuclease sequencing), ChIP-seq (chromatin immunoprecipitation sequencing), and RNA-seq data. Christopher Probert (Stanford University, USA) presented DeepNuc, a deep learning technique able to determine nucleosome positioning from paired-end ATAC-seq datasets. Michael Hoffman (Princess Margaret Cancer Centre, Canada) described a method to analyze ChIP-seq and RNA-seq datasets and classify transcription factor binding sites into four binding variability categories: static, expression-independent, expression-sensitive, and low [9]. Anshul Kundaje (Stanford University, USA) described a deep learning approach that integrates epigenomic datasets (such as DNase-seq or ATAC-seq) to predict transcription factor binding sites across diverse cell types. Kundaje also presented a new way to interpret the learned model (https://github.com/kundajelab/deeplift).

Several presenters described the analysis of transcription factor binding sites and enhancers. Katherine Pollard (University of California, San Francisco, USA) described methods for the analysis and prediction of promoter–enhancer interactions [10]. Vera Kaiser (University of Edinburgh, UK) characterized mutational profiles of transcription factor binding sites. Sarah Rennie (University of Copenhagen, Denmark) presented a Bayesian model across Functional Annotation of the Mammalian Genome (FANTOM) samples to compute simultaneous random walks across sets of potential transcription initiation events. Rani Elkon (Tel Aviv University, Israel) performed a large-scale search for enhancer regions in the human genome [11].

## Data curation and visualization

Speakers described several tools to help genome informaticists to visualize data. Kim Pruitt (National Library of Medicine, USA) described Sequence Viewer to display sequence and annotation data, and Tree Viewer to view phylogenetic tree data. She also presents Genome Workbench (https://www.ncbi.nlm.nih.gov/tools/gbench/), a tool suite that runs both Sequence Viewer and Tree Viewer in local environments. David Powell (Monash University, Australia) presented Degust (http://victorian-bioinformatics-consortium.github.io/degust/), a web tool to analyze gene expression datasets. Degust can produce a principal component analysis visualization, clustering aspects of a user’s dataset. Jonathan Manning (University of Edinburgh) presented Shinyngs (https://github.com/pinin4fjords/shinyngs), an R package for generating plots from RNA-seq data. Birgit Meldal (European Bioinformatics Institute, UK) described the Complex Portal (https://www.ebi.ac.uk/intact/complex/), a tool for analyzing and visualizing protein complexes.

A few speakers presented on curating data from the literature. Alex Bateman (European Bioinformatics Institute, UK) analyzed the feasibility of curating data on biomolecules from the literature. He determined that despite a vast increase in the amount of biomedical literature, most does not need to be analyzed by curators. Benjamin Ainscough (Washington University, St Louis, USA) described DoCM (http://docm.genome.wustl.edu/), a database of known mutations in cancer. DoCM contains approximately 1000 mutations in 132 cell lines.

Ismail Moghul (Queen Mary University of London, UK) presented GeneValidator, which identifies potential problems in gene prediction, by comparing predicted genes with gene annotations from other resources. Ryan Layer (University of Utah, USA) described GIGGLE (https://github.com/ryanlayer/giggle), a fast genomics data search engine.

## Transcriptomics, alternative splicing, and gene prediction

Speakers discussed several aspects of analyzing transcriptomic datasets. Hagen Tilgner (Weill Cornell Medicine, USA) described the use of long read technology to discover novel splice isoforms and long non-coding RNAs (lncRNAs) in the human transcriptome. Simon Hardwick (Garvan Institute of Medical Research, Australia) presented a set of spike-in standards for RNA-seq, called Sequins (http://www.sequin.xyz/). These standards act as a ground truth to measure the accuracy and precision of transcriptome sequencing. Pall Melsted (University of Iceland, Iceland) presented Pizzly, a new tool to detect the gene fusions that often occur in cancer from transcriptome data, approximately 100 times faster than established methods. Annalaura Vacca (University of Edinburgh, UK) presented a meta-analysis of FANTOM5 cap analysis gene expression (CAGE) time-course expression datasets. Using these data, she identified known immediate early genes and candidate novel immediate early genes.

Several speakers discussed new methods for single-cell RNA expression, including scRNA-seq. Davis McCarthy (European Bioinformatics Institute, UK) presented Scater [12], an R package for scRNA-seq datasets. McCarthy stressed the need for carefully designed studies, rigorous quality control, and appropriate handling of batch effects. Alexandra-Chloe Villani (Broad Institute, USA) analyzed chromosomal copy number aberrations and gene expression data on hundreds of individual peripheral blood mononuclear cells. She used Seurat (http://satijalab.org/seurat/) for spatial reconstruction, identifying six subtypes of dendritic cells and respective markers. Johannes Köster (Centrum Wiskunde & Informatica, the Netherlands) a new Bayesian model (http://zhuang.harvard.edu/merfish/) for reducing systematic bias in multiplexed error-robust fluorescence in situ hybridization (MERFISH) data. Shannon McCurdy (University of California, Berkeley, USA) applied a column subset selection method for dimensionality reduction to scRNA-seq datasets. This method selects a subset of columns from a gene expression matrix, preserving properties such as sparsity and interpretability.

## Comparative, evolutionary, and metagenomics

Some projects on analysis of metagenomics datasets were presented. Owen White (University of Maryland, USA) presented an update on the Human Microbiome project, which ties together metagenomics data with phenotype data on host individuals. Curtis Huttenhower (Harvard University) described using HUMAnN2 (http://huttenhower.sph.harvard.edu/humann2) to process metagenomics and metatranscriptome data from the Human Microbiome Project (http://hmpdacc.org/).

A few speakers discussed comparative genomics and evolutionary approaches. James Havrilla (University of Utah, USA) presented a statistical model to identify constraint in different domains within a protein. Sonja Dunemann (University of Calgary, Canada) described the caution necessary before claiming horizontal gene transfer. David Curran (University of Calgary, Canada) presented work on Figmop [13], a profile hidden Markov model that identifies orthologs not identifiable using the popular Basic Local Alignment Search Tool (BLAST) method.

Several speakers described analyses of genetic traits in population-level datasets. Sriram Sankararaman (University of California, Los Angeles, USA) presented an analysis of human admixture with Neanderthal and Denisovan populations [14]. Alicia Martin (Massachusetts General Hospital) presented work using the Sequencing Initiative Suomi (SISu, http://sisuproject.fi/) data to understand recent population history and migration in Finnish populations. Moran Gershoni (Weizmann Institute of Science, Israel) described sex differentially expressed genes from common tissues from Genotype-Tissue Expression (GTEx) [15] data. He identified 244 X-linked sex differentially expressed genes, 16 of which are in multiple tissues.

## Conclusion

The presentations described above were a major attraction of this conference. As in most conferences, of course, the ability to interact with conference attendees provided another major benefit. Increasingly, these benefits accrue not just to the hundreds of in-person attendees at the conference but to thousands of scientists elsewhere. The meeting had an “open by default” policy that encouraged wide discussion of presentations on Twitter and elsewhere. By following the meeting via Twitter, reading preprints on bioRxiv, examining software on GitHub and Bitbucket, and viewing slide decks posted on the internet, many engaged with the advances presented in Hinxton without leaving their home. Even those at the meeting enjoyed an enhanced ability to discuss new work both during and after talks. And those who participated in Twitter found new colleagues to interact and collaborate with long after the meeting ended.

While one can follow Genome Informatics from thousands of miles away, we cannot deny the importance of the meeting itself as a locus for bringing together new research and engaged researchers. Although results are now immediately available to all, there is no substitute for attending in person, which is also the only way to present work at the meeting. And it was the thematically balanced and high-quality program that attracted so much discussion in the first place. We hope that this history of an interesting and excellent scientific program continues and look forward to Genome Informatics 2017.

## Abbreviations

ATAC-seq: Assay for transposase-accessible chromatin followed by sequencing
DoCM: Database of Curated Mutations
MPRA: Massively parallel reporter assay
scRNA-seq: Single-cell RNA sequencing
